# Sleep transcends limited knowledge to support logical reward-related decisions in a novel task in male mice

**DOI:** 10.1186/s13041-025-01267-x

**Published:** 2025-12-13

**Authors:** Mostafa R. Fayed, Khaled Ghandour, Ali Choucry, Kareem Abdou, Kaoru Inokuchi

**Affiliations:** 1 Research Center for Idling Brain Science, University of Toyama, Toyama, Japan; 2 Department of Biochemistry, Graduate School of Medicine and Pharmaceutical Sciences, University of Toyama, Toyama, Japan; 3 Department of Pharmacology and Toxicology, Faculty of Pharmacy, Kafrelsheikh University, Kafr El Sheikh, Egypt; 4 Department of Biochemistry, Faculty of Pharmacy, Cairo University, Cairo, Egypt; 5 Center initiative for training international researchers (CITIR), University of Toyama, Toyama, Japan; 6 Department of Pharmacology and Toxicology, Faculty of Pharmacy, Cairo University, Cairo, Egypt; 7 College of Pharmacy, Al-Ain University, Abu Dhabi, UAE

**Keywords:** Decision-making, Logical, Probability, Safe, Sleep, Idling, Novel task, Deprivation, Reward, Rule

## Abstract

Sleep is essential for strengthening memory and consolidation. Emerging evidence supports its role in cognitive processes such as rule abstraction and inference. However, how sleep influences logical nongambling probabilistic decision-making has yet to be discovered. We developed a reward-based logical decision task that requires rule use and allows scope for reasoning. The mice were able to discriminate between two contexts with different outcomes. This behavior paradigm teaches mice to make free choices between an option to obtain a high-value probabilistic reward in specific entries and a guaranteed safe, low-value option. This knowledge was acquired through six forced entries to each side in training sessions, and they were then tested on subsequent days. As a hidden rule, they may extend their knowledge during these testing sessions by being allowed to take extra entries. We found that extended sleep deprivation disrupted their logical decisions. Sleep-deprived mice were unable to maintain their previous logical performance, resulting in a significant reduction in the rewards they earned. Rule switching in an updated version of the task eliminated gambling-like behavioral dependence in this novel task. These results suggest that adequate sleep is necessary for applying learned knowledge and engaging in complex cognitive functions, such as reasoning.

**Supplementary Information:**

The online version contains supplementary material available at 10.1186/s13041-025-01267-x.

## Introduction

Economic decision-making is a complex cognitive process influenced by multiple factors, such as probability misestimation and loss aversion [1]. Given these conditions, limited knowledge necessitates the use of higher cognitive abilities, such as inductive reasoning, in which initial observations shape a generalized rule-like conclusion [2]. The role of neural reactivations during sleep has been linked to sophisticated cognitive functions such as transitive inference and assimilation of experiences [3–6]. Sleep not only consolidates previous experiences but also prepares a subset of cells for future learning [7, 8]; thus, it is currently considered as an active state contributing to online engagement. Studies on humans and mice indicate that slow oscillations and spindles, predominant during non-rapid eye-movement (NREM) sleep, support memory consolidation, extending the role of sleep to those for learned rules. On the other hand, theta power during rapid eye-movement (REM) sleep promotes rule abstraction and reorganization [6, 9, 10]. Even 15-month-old infants appear to benefit from naps, facilitating abstraction for language learning [11]. The relationship between probabilistic reward and sleep is often studied in the context of risky decision-making in variants of probability discounting and Iowa gambling tasks, where findings are inconsistent [12]. Here, we aimed to design a new paradigm that addresses this relationship but from a nongambling rewarding rule perspective. We developed a minimalist training task that encourages inductive reasoning abilities in mice, which we termed the “logical decision task”.

## Results

**Fig. 1:**
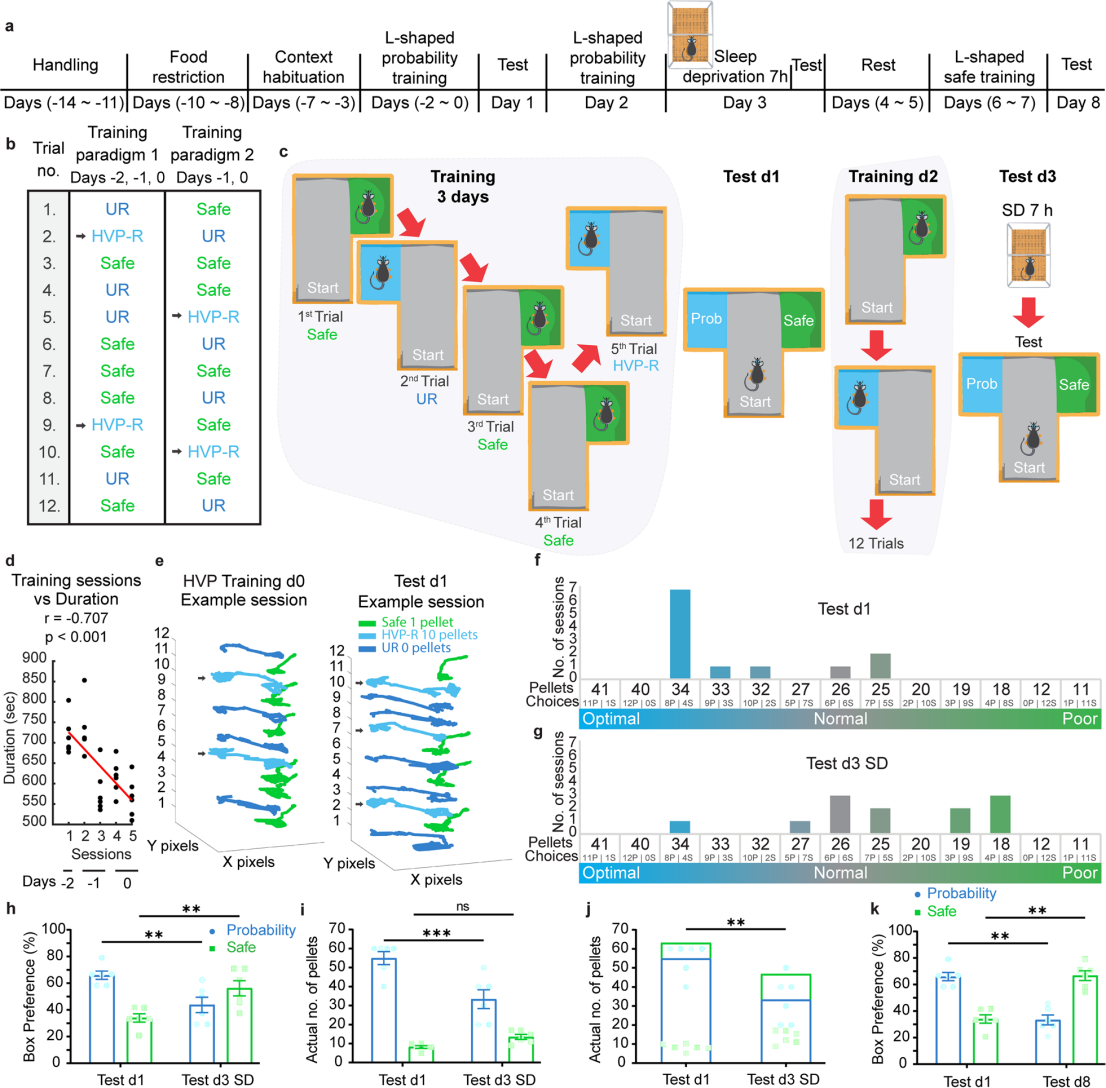
Sleep deprivation impairs performance in the logical decision task. (**a**) The behavioral schedule used to establish the logical decision task in mice. (**b**) The training paradigms employed on training days, where paradigm 1 was used on the first session of training days − 2, − 1, and 0. While paradigm 2 was used on the second session of training days − 1 and 0. The black arrows indicate the second and fifth rewarded entries in the probability box. Safe, HVP-R (High-Value Probabilistic Reward), UR (unrewarded) trials are indicated in green, light blue, and dark blue, respectively. (**c**) Schematic of the training and testing days; SD, Sleep deprivation. (**d**) Scatter plot showing the decline in total training session duration over time. Each dot represents a training session from one mouse (Red line, linear fit; Pearson correlation). (**e**) Actual pose estimation for a representative training and testing session using DeepLabCut software, from start to finish. The black arrows indicate prolonged periods where the animal was consuming ten pellets in the probability box. (**f**,** g**) Representation of the shift in choices between the testing sessions on day 1 (**f**) and day 3 (**g**) among all possibilities. (**h**) Preference for freely made choices over testing days 1 and 3, expressed as a percentage of choosing the probability box or the safe box from total trials for each testing day (two-way RM ANOVA, Šidak’ s multiple comparisons). (**i**) Number of pellets obtained from each box separately (two-way RM ANOVA, Šidak’ s multiple comparisons). (**j**) Total pellets collected during the entire testing days (paired t-test). (**k**) Preference for freely made choices over testing days 1 and 8, expressed as a percentage of choosing the probability or the safe box from total trials, indicating a shift toward safe choices (two-way RM ANOVA, Šidak’ s multiple comparisons). *n* = 6. **P < 0.01; ***P < 0.001; ns, not significant (*P* > 0.05). The data are presented as means ± SEM

We spent just under two weeks preparing the mice for the upcoming training. First, mice were acclimated to being handled through integrated cupping and tunneling methods, as handling mice by their tails might impair their behavioral performance [13]. This was followed by initiating food restriction before proceeding to stepwise habituation to the T-maze (Fig. 1a). This preparatory segment aimed to reduce stress and eliminate factors that might interfere with the animals’ ability to focus on grasping the subsequently introduced reward rule. To establish the “logical decision task”, animals needed to learn to distinguish between two contextual options with different reward outcomes and then choose their preferred context logically during the test. Each training session consisted of 12 single trials where the mice encountered an L-shaped entrance leading to either the safe box, where they received a guaranteed single pellet, or the probability box, which offered a one-third probability of a larger reward, making the probability box more favorable in terms of the total number of pellets received (Fig. 1b). A minimalist training paradigm was employed, consisting of 60 trials divided into five sessions over three days. Different trial sequences were presented on two training paradigms to eliminate mice relying on trial’s order; instead, they needed to learn the common rule that they are rewarded on the second and fifth entries to the probability box (see Methods; Fig. 1b). They, in some sense, needed to count entries and recognize that the unrewarded trial in the probability box was a step toward reaching the rewarded one. The mice were trained on six entries to each box, with a hidden rule allowing them to choose either box during a test session of 12 entries (Fig. 1c). Task rule understanding was evidenced by the decreasing duration of successive training sessions (Fig. 1d). This schedule prevented overtraining and created an opportunity for the mice to excel in subsequent testing sessions based on their reasoning abilities. In this task, mice were trained to expect a high-value probabilistic reward (HVP-R) only on the second and fifth trials. However, during the test session (12 trials), they were allowed to extend this training and also obtain high-value reward on the eighth and eleventh trials, thereby reaching the optimal number of pellets, hence the hidden rule.

To assess the importance of sleep in comprehending this task, we aimed to test the effect of sleep deprivation on reasoning ability in terms of the total reward pellets earned (Fig. S1a). Task understanding was evident (Fig. S1b), and we found that four hours of sleep deprivation on test day 3 was not sufficient to alter their initial testing preference on test day 1 (Fig. S1c and d). The mice mostly preserved the same performance across both testing days. To further clarify the effect of sleep on task performance, we repeated the experiment with two modifications: first, we doubled the number of reward pellets in the probability box (ten pellets instead of five) to make that choice more appealing; second, we increased sleep deprivation to seven hours for better separation from the noneffective four-hour duration (Fig. 1a-e). By plotting the number of pellets based on all possible choices in the test session, the optimal performance on test day 1 declined on test day 3 to levels falling below their normal reward rule on training days (Fig. 1f and g). The preference for the probability box significantly decreased after sleep deprivation test day 3 compared with the initial test day 1 (Fig. 1h). The total number of pellets obtained from the probability box or both boxes combined also decreased significantly (Fig. 1i and j). These results highlight the necessity of sleep in grasping and later applying this hidden rule to acquire larger rewards.

To further clarify the objective of the task, we performed a rule switch. In brief, the mice were again trained on days six and seven with a simple but decisive change in the probability box rule; the number of pellets obtained on rewarded trials in the probability box became two pellets instead of ten, making the total number of pellets received in the probability box less than that in the safe box while maintaining the same trial sequences from the original training paradigms (see Methods; Fig. 1a). This transformed the probability box into a lower-value choice, and testing on day 8 assessed the effectiveness of the new rule. The shift in favorability toward the safe box resulted in a behavioral shift in preference, with the mice moving away from the new probabilistic (lower-value) option (Fig. 1k). This shift also indicated that the mice were not pursuing a risky option but rather a calculated rewarding rule. Overall, mice can attempt to discover the hidden reasoning rule to obtain a larger reward; nevertheless, sleep deprivation appears to impair this ability.

## Discussion

We developed a novel “logical decision task” that assessed rule acquisition and inductive reasoning, where logical decisions were disrupted by sleep deprivation. Studies have shown that sleep helps integrate visual and auditory cues, influences cognitive flexibility, and increases creativity [4, 14]. For example, targeted memory reactivation of auditory cues during REM sleep improved rule abstraction in a reasoning task [9]. However, gambling-related studies on rodents that focused on risky decision-making revealed a negligible effect of sleep deprivation on preferences for advantageous choices [12, 15]. Accordingly, the change in preference in our task after sleep deprivation is likely to result from a decline in logical ability due to failure in task rule retrieval and reasoning rather than gambling-like behavior, which was further confirmed by the rule switch. This shift in favorability also suggests that the rule-based preference with hidden contingencies is more likely to explain the choices of the mice than the innate probabilistic bias. The animals were allowed to generalize the six example-probability box entries rule observed during training and infer the hidden rule for the other six entries during testing. This hidden rule enables the animal to make analogies, where initial observations influence the likelihood of its outcome [2]. Our findings showed that mice could extend beyond their learned knowledge to solve the current problem, but this ability was limited by poor sleep. Moreover, sleep deprivation may have also affected the mice’s motivation to perform the challenging probabilistic assessment in this task. Collectively, these results suggest that sleep is vital for both abstracting the hidden rule and later its implementation.

We designed this task to create a temporal separation between the learning and testing timepoints, with each conducted on distinct days; this separation is mostly occupied by sleep and quiet wakefulness, collectively termed “idling states” [3]. Further research is needed to explore the neural dynamics of the brain regions involved in this logical decision-making process during idling states.

## Methods

### Animals

All the mice used in this study were naïve wild-type male C57BL/6 mice purchased from Sankyo Labo Service Co. Inc. (Tokyo, Japan). They were maintained on a 12 h light/dark cycle at 24 ± 3 °C and 55 ± 5% humidity. All the behavioral sessions were performed during the light period between zeitgeber ZT + 6 and ZT + 12. The mice used in the behavioral experiments were 12 to 20 weeks old. The mice were housed individually in a microisolation rack system (FRP BIO2000, CLEA Japan) that comprised 16 individually ventilated boxes with glass-fiber filters. To prevent any disturbance in sleep patterns, the mice were individually housed in each box at the beginning of the behavioral protocol. The mice were randomly assigned to experimental conditions.

## Logical decision task

The T-maze consisted of 3 physical acrylic contexts: a start-choice arm (305 mm length × 150 mm width × 200 mm height) and [2] side-open decision boxes (185 mm length × 150 mm width × 200 mm height). The starting-choice context is a gray acrylic floor separated at the midline by a gray acrylic 5 mm wide manual vertical lift door. This separation physically divides the context into a start box and a choice box opened from both left and right. The first decision context is blue with a green hard rubber floor of pointy texture. The second one is transparent on the 2 sides opposite each other, while the wall facing the entrance has a black vertical lines pattern on a white background; the floor of this context is hard black polypropylene. Both decision boxes can be closed with an acrylic manual vertical lift door of the same color/pattern as the context. A modification was made to the maze before the start of the project; we raised the total height of the entire T-maze to 250 mm in height with transparent polypropylene. Innate bias among the mice was eliminated by exchanging the decision boxes, which were probability or safe for each mouse. Additionally, both boxes were exchanged between the mice, being on the right or the left.

The behavioral protocol consists of 3 segments: a preparatory segment ending on day − 3 (handling, food restriction, and habituation), an L-shaped training segment, and a testing segment starting from day 1 (Fig. 1a and S1a).

*Handling*: This phase consisted of 4 days; its purpose was to relieve stress that might cause the mouse to be distracted from the upcoming learning. We chose to handle mice via an optimized method that incorporates handling through the tunnel (mouse tunnel K3323, AS ONE Japan) and cupping. On the first day, we introduced a tunnel in the middle of the home cage for 5 min. Then, we instructed the mouse by hand to enter the tunnel to be lifted and dropped backward to be cupped in the hand for 6 min. On the following 3 days, the handling session lasted for 6 min, and it included the exchange of the mouse between both hands and through the tunnel. On the 3rd day of handling, we introduced the reward dish (Culture Dish 353001, Falcon) to the home cage. After the handling session on the 4th day, we recorded the weight of each mouse and added only a weight-adjusted normal food pellet to decrease the weight to 80% of the original weight.

*Food restriction*: This phase lasted for 3 days so that the mouse would not associate the initial suffering due to food restriction with the task. The food pellet was adjusted daily for the mouse to reach the desired weight, starting with a food pellet that represented 6% of the original weight. This pellet adjustment would continue throughout the behavioral protocol afterwards.

*Habituation*: This phase consisted of 5 days; the first 2 days consisted of habituation to each box of the 4 boxes separately for five minutes each. For the habituation session for the next two days, the mouse was habituated to either of the L-passages to the decision boxes for 10 min each. On the final day, all the doors were opened, and the mouse explored the full T-maze freely for 10 min starting from the start box. From the 2nd habituation day, after the habituation session was finished, 20 reward pellets (Dustless Precision Pellets 14 mg F05684, Bio-Serv) were introduced in succession inside a reward dish in the home cage.

*L-shaped training*: This phase consisted of 3 days, representing five training sessions. Despite the presence of two forced training paradigms, both had a common rule; that is, in the probability box, the mouse received five reward pellets (Fig. S1) or ten reward pellets (Fig. 1) on the 2nd and 5th entries to the probability box specifically. Starting from this phase, we put 1 reward dish in the safe box and 5 reward dishes in the probability box, where the reward pellet/s would be introduced manually. The mouse learned that the choice was made when it arrived and sniffed the reward dish. We started the session by putting the mouse in the start box with the door closed; then, upon lifting the door, the mouse would have no option except for the L-shaped entrance to the open box. If it was a safe trial, the mouse would receive a reward pellet by hand in the reward dish present in the safe box. After eating, the mouse was returned to the starting box via the cupping handling method to initiate the next trial. If it was an UR trial, the mouse would receive no reward pellets in the probability box, and the trial would be considered finished. If it was a HVP-R trial, the mouse would receive five (Fig. S1) or ten (Fig. 1) reward pellets in the probability box.

In the four-hour sleep deprivation experiment (Fig. S1), the 1st training paradigm, which was conducted on the 1st, 2^nd,^ and 3rd days, was in the following sequential trial order: UR 0, HVP-R 5, safe 1, UR 0, UR 0, safe 1, safe 1, safe 1, HVP-R 5, safe 1, UR 0, safe 1. Numerals indicate the number of reward pellets. The 2nd training paradigm, conducted on the 2nd and 3rd days after approximately 90 min of the 1st training paradigm session, was in the following sequential trial order: safe 1, UR 0, safe 1, safe 1, HVP-R 5, UR 0, safe 1, UR 0, safe 1, HVP-R 5, safe 1, UR 0.

In the seven-hour sleep deprivation experiment (Fig. 1), the 1st training paradigm was in the following sequential trial order: UR 0, HVP-R 10, safe 1, UR 0, UR 0, safe 1, safe 1, safe 1, HVP-R 10, safe 1, UR 0, safe 1. The 2nd training paradigm was in the following sequential trial order: safe 1, UR 0, safe 1, safe 1, HVP-R 10, UR 0, safe 1, UR 0, safe 1, HVP-R 10, safe 1, UR 0.

We intentionally aimed that mice do not depend on the trial’s order of receiving the first and second HVP-R being on 2nd /5th or 9th/10th trials, respectively. But rather depend on the shared rule between the two paradigms, which is that the HVP-R is exclusively supplied on the second and fifth entries to the probability box. We chose the second entry since this entry is situated at the midpoint.

Our inclusion criterion for this task was that any mouse that completes the training and fully consumes the pellets supplied during the training would be subjected to the test. Despite the variable duration of learning, all mice fulfilled this criterion and were included, and they were all subjected to the test sessions.

*The testing segment* (Fig. S1): This segment starts 1 day after the aforementioned “last training day”. Day 1 represented test day 1, where the mouse was tested for the probability-favored rule taught after normal sleep in 2 sessions, with 12 trials each. The session started by putting the mouse through cupping in the closed start box while both decision boxes were left open. Upon lifting the door of the starting box, the mouse would have two options. If it chose the safe box by stopping at the dish, it would receive a single reward pellet. If it chose the probability box, it would receive 5 reward pellets, not only on the 2nd and 5th entries as it has learned but also on the 8th and 11th entries as a hidden rule. If the entrance to the probability box was any other than the previously mentioned entries, the mouse would not receive any reward pellets. Day 2 was another training day for two sessions to reinforce the rule. Day 3 represented test day 3, when the mouse was tested just as on test day 1; with a modification, they were subjected to sleep deprivation for four hours by gently touching the home cage just before the test.

*The testing segment* (Fig. 1): Days 1, 2, and 3 were exactly similar to those previously described, except that the number of reward pellets in the probability box was ten instead of five, and the duration of sleep deprivation on day 3 was seven hours. On days 4 and 5, the mouse rested in the home cage, where it was not engaged in any behavioral session.

*Safe training paradigm* (Fig. 1k): Days 6 and 7 represented the forced L-shaped entrance training for the newly introduced safe-favored rule with only 2 reward dishes in the same probability box. In this updated behavioral paradigm, mice received 2 reward pellets as a low-value probabilistic reward (LVP-R). The 1st training paradigm conducted on days 6 and 7 was in the following sequential trial order: UR 0, LVP-R 2, safe 1, UR 0, UR 0, safe 1, safe 1, safe 1, LVP-R 2, safe 1, UR 0, and safe 1. The 2nd training paradigm, conducted on the 7th day after the 1st training paradigm, is in the following sequential trial order: safe 1, UR 0, safe 1, safe 1, LVP-R 2, UR 0, safe 1, UR 0, safe 1, LVP-R 2, safe 1, and UR 0. The mouse was trained such that the safe box was slightly better than the updated probability box. Day 8 represented test day 8, when the mouse was tested for the updated safe-favored rule in the same manner as on test day 1.

## Behavioral analysis

Behavioral sessions were recorded via an overhead web camera (HD pro C920, Logitech) mounted on a vertical stand. The box preference is calculated as follows:

$$ \begin{gathered} Box\:preference \hfill \\ = (Preference_{x} /Preference_{T} )\: \times \:100 \hfill \\ \end{gathered} $$

where $\:x$ is either the probability or the safe box, and where $\:T$ is the total number of trials.

For the example sessions (Fig. 1e), the animal pose estimation software DeepLabCut [16] was used to extract the x–y coordinates of the mouse during the sessions. We considered the trial starting time point from the moment the mouse was in the start box and the ending time point with the full consumption of pellet/pellets or arrival at the dish, and was not supplied with the reward in UR trials.

### Statistics

Statistical analyses were performed via Prism 9 (GraphPad Software) and MATLAB (MathWorks). The statistical analyses used are presented in each panel legend and a detailed table within “Additional file 1”.

## Supplementary Information

Below is the link to the electronic supplementary material.

Supplementary Material 1

## Data Availability

The datasets used and/or analyzed during the current study are available from the corresponding author on reasonable request.

## Abbreviations

HVP-R: High-value probabilistic reward
LVP-R: Low-value probabilistic reward
NREM: Non-rapid eye-movement
REM: Rapid eye movement
RM: Repeated measures
SD: Sleep deprivation
UR: Unrewarded
ZT: Zeitgeber
