# Supplementary material for: Sleep transcends limited knowledge to support logical reward-related decisions in a novel task in male mice
Source: Mol Brain. 2025 Dec 13;19:6. doi: 10.1186/s13041-025-01267-x (PMC12821788; doi:10.1186/s13041-025-01267-x)
Supplement: Supplementary file 1 — Supplementary Material 1 [file 13041_2025_1267_MOESM1_ESM.docx]

**Additional file 1**

**Sleep transcends limited knowledge to support logical reward-related decisions in a novel task in male mice**

**Mostafa R. Fayed^1,2,3^, Khaled Ghandour^1,2,4,5^, Ali Choucry^1,2,6^, Kareem Abdou^7^, Kaoru Inokuchi^1,2*^**

*Correspondence:

Kaoru Inokuchi

[inokuchi@med.u-toyama.ac.jp](mailto:inokuchi@med.u-toyama.ac.jp)

**Supplementary Figure**


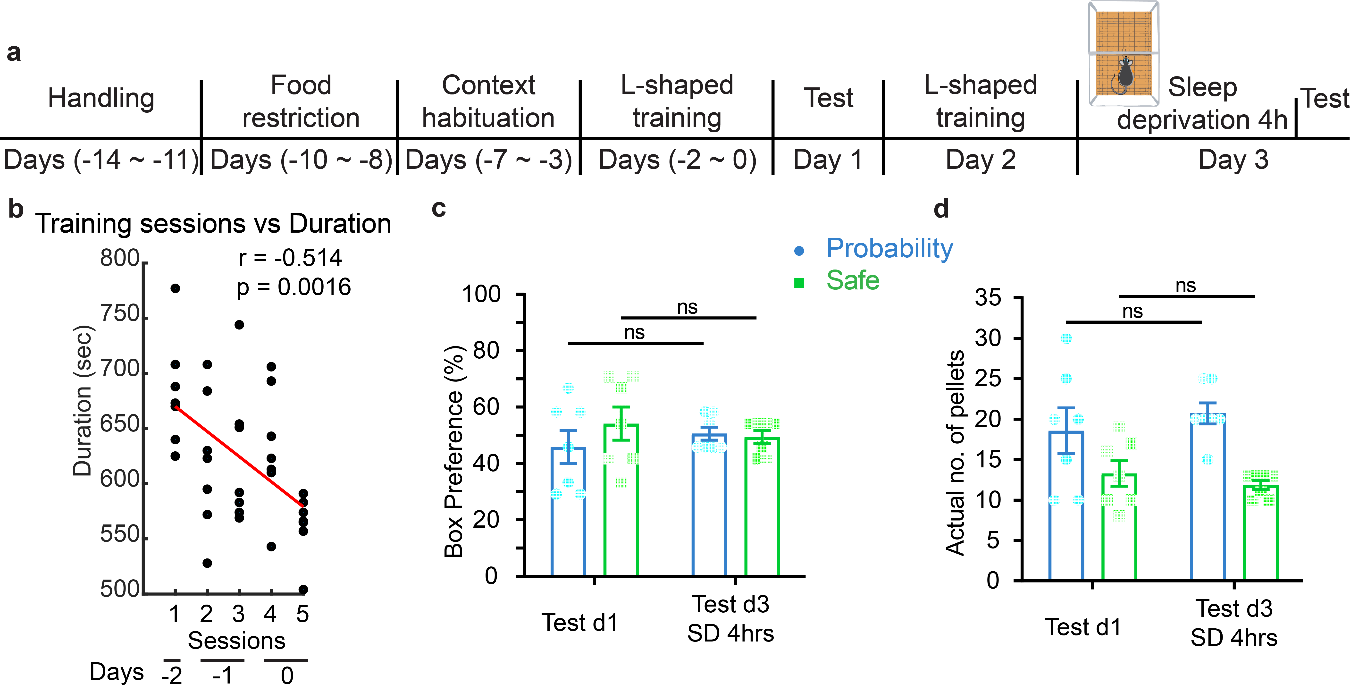


**Fig. S1** Four-hour sleep deprivation is insufficient to alter logical performance. **a** The behavioral schedule for the logical decision task in mice. **b** Scatter plot showing the decline in total training session duration over time. Each dot represents a training session from one mouse (Red line, linear fit; Pearson correlation). **c** Preference for freely made choices over testing days 1 and 3, expressed as a percentage of choosing the probability or the safe box out of total trials for each testing day (two-way RM ANOVA, Šidak' s multiple comparisons). **d** Number of pellets obtained from each box separately (two-way RM ANOVA, Šidak' s multiple comparisons). n = 7. ns, not significant (P > 0. 05). The data are presented as means ± SEM.

**Details of statistical analyses**

| **Figure** | **Sample Size** | **Panel** | **Analysis** | **Statistical Test Values** |
| --- | --- | --- | --- | --- |
| **Fig. 1** | n = 6 mice | 1d | Pearson Correlation | r = -0.7069, p = 1.258e-05 |
|  |  | 1h | Two-way RM ANOVA Row Factor: Testing days Column Factor: Probability-Safe Post-hoc test: Šidak`s multiple comparisons test | Interaction: *F* (1, 5) = 49.23, P = 0.0009 Šidak`s multiple comparisons test: ** P = 0.0085 |
|  |  | 1i | Two-way RM ANOVA Row Factor: Testing days Column Factor: Probability-Safe Post-hoc test: Šidak`s multiple comparisons test | Interaction: *F* (1, 5) = 55.51, P = 0.0007 Šidak`s multiple comparisons test: Probability: *** P = 0.0008, Safe: P = 0.1754 |
|  |  | 1j | Paired t-test Two-tailed | t = 6.213, ** P = 0.0016 |
|  |  | 1k | Two-way RM ANOVA Row Factor: Testing days Column Factor: Probability-Safe Post-hoc test: Šidak`s multiple comparisons test | Interaction: *F* (1, 5) = 49.98, P = 0.0009 Šidak`s multiple comparisons test: ** P = 0.0082 |
| **Fig. S1** | n = 7 mice | 1b | Pearson Correlation | r = -0.5142, p = 0.0016 |
|  |  | 1c | Two-way RM ANOVA Row Factor: Testing days Column Factor: Probability-Safe Post-hoc test: Šidak`s multiple comparisons test | Interaction: *F* (1, 6) = 0.9321, P = 0.3716 Šidak`s multiple comparisons test: P = 0.7698 |
|  |  | 1d | Two-way RM ANOVA Row Factor: Testing days Column Factor: Probability-Safe Post-hoc test: Šidak`s multiple comparisons test | Interaction: *F* (1, 6) = 0.8322, P = 0.3968 Šidak`s multiple comparisons test: Probability: P = 0.7173  Safe: P = 0.8588 |
